# *H. pylori* Eradication Treatment Causes Alterations in the Gut Microbiota and Blood Lipid Levels

**DOI:** 10.3389/fmed.2020.00417

**Published:** 2020-08-11

**Authors:** Gracia M. Martín-Núñez, Isabel Cornejo-Pareja, M. del Mar Roca-Rodríguez, Mercedes Clemente-Postigo, Fernando Cardona, José C. Fernández-García, Isabel Moreno-Indias, Francisco J. Tinahones

**Affiliations:** ^1^Unidad de Endocrinología y Nutrición, Hospital Universitario Virgen de la Victoria, Málaga, Spain; ^2^Centro de Investigación Biomédica en Red de Fisiopatología de la Obesidad y la Nutrición (CIBEROBN), Instituto de Salud Carlos III, Madrid, Spain; ^3^Departamento de Endocrinología y Nutrición, Hospital Universitario Puerta del Mar, Cádiz, Spain; ^4^Departamento de Biología Celular, Fisiología e Inmunología, Instituto Maimónides de Investigación Biomédica de Córdoba (IMIBIC), Universidad de Córdoba, Hospital Universitario Reina Sofía, Córdoba, Spain

**Keywords:** LDL, HDL, gut microbiota, antibiotic, *H. pylori*

## Abstract

**Background:** The gut microbiome plays an important role in the lipid metabolism. Antibiotic treatment causes changes in the intestinal microbiota. Our objective was to explore the relationship between changes in the intestinal microbiota and the level of plasma high density lipoprotein cholesterol (HDL) and low density lipoprotein cholesterol (LDL).

**Methods:** Prospective case-control study with *Helicobacter pylori*-positive patients undergoing eradication therapy with omeprazole, clarithromycin, and amoxicillin. Stool and blood samples were obtained from 20 controls (*H. pylori* negative) and 40 patients before and 2 months after antibiotic treatment. Gut microbiota was determined through 16S rRNA amplicon sequencing (Illumina MiSeq).

**Results:** Eradication treatment for *H. pylori* increased the HDL levels, and caused changes in gut microbiota profiles. An unfavorable lipid profiles (high LDL and low HDL levels) was associated with a low microbial richness and an increase of the Bacteroidetes phylum. *Prevotella copri, Lachonobacterium*, and *Delsufovibrio* were positively associated with HDL while *Rikenellaceae* was negatively associated with HDL after completing antibiotic treatment.

**Conclusions:** *Helicobacter pylori* eradication treatment could improve lipid metabolism in relation with an increase in the HDL. Changes in the abundance of specific bacteria, such as *P. copri, Lachonobacterium, Delsufovibrio*, and *Rikenellaceae* could be associated with change in the plasma HDL levels.

## Introduction

Increasing evidence has demonstrated that the intestinal microbiota is critical for the development of diseases associated with altered lipid metabolism (1) including variation in the level of blood lipids (2). The microbiome affects ability to metabolize lipids by the host taking part in the absorption, storage, and energy resulted from the diet (3), because of its ability to convert bile acids and produce short-chain fatty acids (SCFAs) within the intestinal lumen (4, 5).

Triglycerides and high density lipoproteins cholesterol (HDL) levels have been associated to the diversity and amount of Proteobacteria and Bacteroidetes members (including *Christensenellaceae, Pasteurellaceae*, and genus *Butyricimonas*) (2). HDL function is to transport cholesterol to the liver from peripheral tissues for its subsequent excretion as biliary sales, and to endocrine organs for the synthesis of steroid hormones. The concentration of serum low-density lipoproteins cholesterol (LDL) is regulated, in part, by a cross-talk between the absorption of dietary and biliary cholesterol in the intestine, and the biosynthesis of cholesterol in the liver (6). LDL and HDL levels are biomarkers associated with the CVD risk (7).

*Helicobacter pylori* is a Gram-negative bacterium that colonizes the gastric mucosa commonly causing a chronic infection. Also, it has been associated with extradigestive pathologies including coronary heart disease (8). *H. pylori* eradication through oral administration of antibiotic and a proton-pump inhibitor has been associated with alterations in the gut microbiota (7, 9). We have previously found that antibiotic therapy used for *H. pylori* eradication can alter the intestinal microbiota population, and more importantly, these changes were associated to glucose metabolism and GLP-1 (10, 11). However, there is scarce data in humans about the relationship between blood lipids and changes in the profile of gut microbiota after antibiotic therapy (12). Thus, in the present study, our objective was to test the relationship between changes in the gut microbiota and blood lipids level in patients who received antibiotic treatment for *H. pylori* eradication.

## Materials and Methods

### Study Subjects and Design

Forty volunteers with positive *H. pylori* antigen in faeces tested by immunochromatography were derived from the Microbiology Unit of the Virgen de la Victoria Hospital (Málaga, Spain). Sample size was calculated taking into account a reduction in richness (according to Chao1 index) of 16% after the antibiotic therapy (13, 14). The inclusion criteria were: (1) adulthood (18–65 years old), and (2) primary infection with *H. pylori*. Besides, a group of non-infected volunteers (20 individuals) was included as control. Exclusion criteria were (1) diabetes (type 1 or 2); (2) prior H. *pylori* documented treatment; (3) antibiotherapy 3 months before recruitment; and (4) without informed consent.

The study included two visits, before and 60 days post-treatment (20 mg omeprazole, 500 mg clarithromycin, and 1000 mg amoxicillin, twice daily during 10 days) for patients, while a unique visit was assessed in the case of the control volunteers. Visitations comprised a physical exploration, a fasting blood sample, and an oral glucose tolerance test (OGTT) with 75 g glucose and measurements at 30, 60, and 120 min. Moreover, samples of stool were obtained in every visit and immediately frozen at −80°C. The investigation protocol was conducted consonantly with the Declaration of Helsinki and conveniently approved by the Medical Ethics Committee at Virgen de la Victoria University Hospital. Volunteers were supplied with a written informed consent, as well as they were advised of the study characteristics.

### Anthropometric, Biochemical, and Dietetics Measurements

Anthropometric measurements (height, weight, and waist and hip circumferences) were recorded (15). An enzymatic method was used for the measurement of triglycerides (mmol/L), total cholesterol (mg/dl), and HDL (mg/dl) (Randox Laboratories Ltd.), while C-reactive protein (CRP) was measured with a Dimension autoanalyzer (Dade Behring Inc.). LDL (mg/dl) was determined through the Friedewald formula. Food intake was evaluated with 7/24-h dietary recalls. The dietary variables (fats, fiber, proteins, etc.) were determined using DIAL nutrition software and the Diet Balancer software (Cardinal Health Systems Inc.).

### Gut Microbiota Analysis

The determination of the gut microbiota was assessed as was described previously (11). In brief, the QIAamp DNA Stool Mini Kit was used for the extraction of DNA from fecal samples, and posteriorly the DNA concentrations were determined by Qubit® Fluorometric (Thermo Fisher Scientific). The 16S rRNA V3-V4 amplicon was analyzed with the universal primers reported by Klindworth et al. (16). The amplicon size ~460 bp was verified with a bioanalyzer (Agilent 2100, USA). AMPure XP beads (Beckman Coulter Genomic, CA, USA) were used to purify amplicon products. Samples were multiplexed with Nextera XT Index Kit (Illumina, CA, USA). Illumina MiSeq platform (Illumina, San Diego, USA) was used for the paired-end sequencing of amplicons.

Quantitative Insights into Microbial Ecology (Qiime) tool (version 1.9.1, open source software) was used for the analysis of the merged paired-end reads. The operational taxonomic units (OTUs) were generated at 97% similarity and alignment by UCLUST consensus using the Greengenes 16S rRNA gene database. Alpha diversity (Chao1 and Shannon indixes, as well as the observed species) was also assessed with Qiime. Alpha diversity was evaluated with the rarefaction workflow, with a threshold of 34,385 sequences. Rarefied data were used for the downstream analysis. OTUs in less than five different samples were excluded. Raw data is sored at the public repository SRA database (NCBI) with the BioProject PRJNA517270.

### Statistical Analysis

SPSS 22.0 (SPSS Inc., Chicago, IL, USA) and QIIME (version 1.9.1; open source software) were used for the statistical analysis. The data were expressed as mean ± standard deviation. Statistical comparisons between the means for independent samples and paired samples (before and after antibiotic eradication treatment) was performed using the Student's *t*-test. Mann-Whitney and Wilcoxon signed-rank tests were used for the evaluation of non-parametric variables. Correlation analysis between analytical, clinical, and microbial populations variables was analyzed using the Spearman bivariate correlations test. Linear regression models were applied to identify bacterial changes as independent predictors of the selected variables (HDL/LDL ratio and HDL–). A *p* < 0.05 treshold was established for the statistical significance.

## Results

### Lipid Metabolism

The anthropometric and clinical variables of the patients and control subjects have been previously described (11). Anthropometric variables as BMI and waist circumference or biochemical parameters as triglycerides and cholesterol level did not reach statistical differences between groups. Focusing on lipid metabolism, HDL levels increased significantly after *H. pylori* eradication with antibiotic therapy (*p* = 0.021), while LDL levels were significantly lower in controls than in *H. pylori*-infected subjects (*p* = 0.036; Table 1). In addition, HDL/LDL ratio was significantly lower in patients pre- and post-*H. pylori* eradication than in control subjects (*p* = 0.007 and *p* = 0.03, respectively; Table 1). HDL/LDL ratio and HDL were associated with BMI in a regression model (*R*^2^ = 0.079, β = −0.281, *p* = 0.005; *R*^2^ = 0.93, β = −0.305, *p* = 0.003, respectively). No statistically differences in the dietary intake energy, micro or macronutrients and dietary fiber between patients and controls (*p* > 0.05) (data not shown).

**Table 1:** Characteristics of the study groups.

**Variables**	**Pre-*H. pylori* eradication (*n* = 40)**	**Post-*H. pylori* eradication (*n* = 40)**	**Control group (*n* = 20)**
Age (years)	46.95 ± 12.78	46.95 ± 12.78	43.86 ± 12.63
Men/women (*n*)	16/24	16/24	9/13
BMI (kg/m^2^)	26.92 ± 4.30	26.91 ± 4.40	25.89 ± 4.54
Waist (cm)	92.10 ± 12.06	91.27 ± 11.73	89.8 ± 13.23
HDL (mg/dL)	52.97 ± 12.9^a^	55.36 ± 16.36^a^	57 ± 15.8
LDL (mg/dL)	121.45 ± 35.8^a^	117.96 ± 33.4	102.05 ± 34^a^
HDL/LDL	0.47 ± 0.17^b^	0.51 ± 0.22^a^	0.62 ± 0.25^b, a^
Triglycerides (mg/dL)	97.2 ± 39.6	93.5 ± 36.4	89.70 ± 41.78
Cholesterol (mg/dL)	194.22 ± 40.84	191.34 ± 37.15	177.05 ± 39.5
DBP (mmHg)	77.75 ± 9.58	80.50 ± 11.37	75.95 ± 10
SBP (mmHg)	123.84 ± 16.62	125.42 ± 21.36	120.3 ± 13.35
CRP (mg/L)	4.07 ± 2.44	3.56 ± 2.11	4.14 ± 2.92

*All values are means ± standard deviations. Wilcoxon's test was used to compare patients before and after H. pylori eradication. The Mann-Whitney U-test or Student's t-test were used to compare independent group. Equal letter indicates statistical signiticative differences in the mean between those 2 groups, p < 0.05. BMI, body mass index; HDL, high-density lipoprotein; LDL, low-density lipoprotein; TGs, triglycerides, TC, total cholesterol; DBP, diastolic blood pressure; SBP, systolic blood pressure; CRP, C-reactive protein*.

### Gut Microbiome and Lipids Metabolism

Our *H. pylori*-positive patients study model who received antibiotics for a limited period of time has allowed us to evaluate changes in the microbiota, HDL and LDL, avoiding the confounding factors derived from the presence of other diseases, or metabolic alterations.

*Helicobacter pylori* infection and its eradication with antibiotic affected alpha diversity (Chao 1 and Shannon indexes) and this data has been previously described by our group (11). Control subjects showed a greatest diversity and richness, with statistically significant differences, compared to the *H. pylori* patients (pre- and post-eradication treatment) (*p* < 0.05) (11). Chao1 index correlated positively with the HDL/LDL ratio (*r* = 0.258, *p* = 0.04).

**Figure 1:**
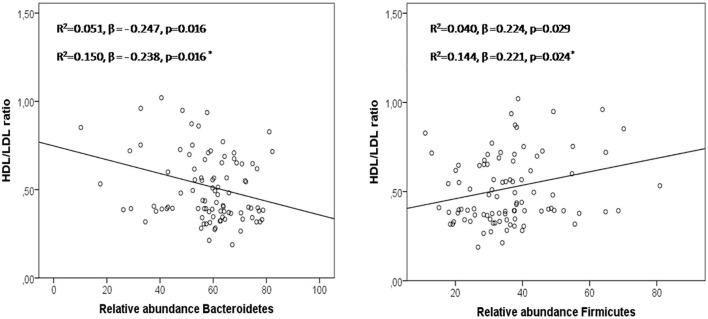
Bacteroidetes and Firmicutes in the prediction of HDL/LDL ratio in a linear regression model including the 3 groups studied. Asterisk: regression model adjusted by age, sex, and BMI.

According to the abundance of each identified OTU, the most abundant bacterial phyla were Firmicutes and Bacteroidetes, while Actinobacteria, Proteobacteria, and Verrucomicrobia contributed with smaller proportions (1–5%). Changes in the microbiome between these subjects has been described in detail by our group in a previous work (11). The phylum Bacteroidetes, that was greater in patients (58.72 ± 13.62 and 63.50 ± 10.30 vs. 45.89 ± 13.57), showed negative correlations with the HDL/LDL ratio (*r* = −0.237, *p* = 0.021), while the phylum Firmicutes, which was greater in controls (45.68 ± 15.61 vs. 35.29 ± 11.82 and 32.06 ± 10.29, *p* < 0.05), showed positive correlations with the HDL/LDL ratio (*r* = 0.230, *p* = 0.025) in the studied groups. In the regression analysis, Bacteroidetes and Firmicutes predicted changes in the HDL/LDL ratio (Figure 1).

Genera and species of the phyla Bacteroidetes and Firmicutes showed association with the HDL/LDL ratio (Table 2). *Bacteroides coprophilus, Eubacterium*, and *E. biforme* predicted changes in the HDL/LDL ratio, in a linear regression model adjusted for age, sex, and BMI (Table 2). The presence of these bacteria was higher in subjects with *H. pylori* infection compared to controls (*p* < 0.05, data not shown). The associations shown in the Table 2 were found in patients and controls.

**Table 2:** Association between intestinal bacteria and HDL/LDL ratio.

**Genus/Species**	**Model 1**			**Model 2**		
	***R*^**2**^**	**β**	***p***	***R*^**2**^**	**β**	***P***
*B. coprophilus*	0.049	−0.243	0.018	0.148	−0.233	0.018
*Eubacterium*	0.043	−0.231	0.025	0.148	0.235	0.018
*E. biforme*	0.045	−0.236	0.021	0.149	−0.237	0.017

*Dependent variable: HDL/LDL ratio. Model 1: linear univariate regression model. Model 2: linear multivariate regression model adjusted by age, sex, and BMI*.

**Figure 2:**
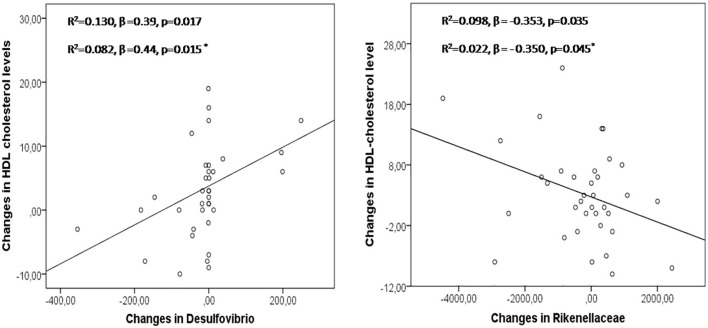
Changes in *Desulfovibrio* and *Rikenellaceae* in the prediction of modifications in HDL levels after *H pylori* eradication treatment. Asterisk: linear regression model adjusted by age, sex, and BMI.

We evaluated the impact of antibiotic eradication therapy on the abundance of identified OTU, LDL, and HDL level. Significant correlations between changes in the number of particular bacteria and HDL were found after *H. pylori* eradication (*Prevotella copri*: *r* = 0.34, *p* = 0.037; *Prevotella stercorea*: *r* = −0.34, *p* = 0.04; *Lachnobacterium r* = 0.36 *p* = 0.028; *Delsufovibrio r* = 0.48, *p* = 0.003). Changes in the abundance of *Bacteroides* were associated with changes in HDL/LDL ratio after *H. pylori* eradication (*r* = −0.364, *p* = 0.029). In multivariate regression analysis, changes in *Delsufovibrio* (*R*^2^ = 0.130, β = 0.39, *p* = 0.017, and *R*^2^ = 0.082, β = 0.44, *p* = 0.015, after including age, sex, and BMI) and *Rikenellaceae* (*R*^2^ = 0.098, β = −0.353, *p* = 0.035, and *R*^2^ = 0.022, β = −0.350, *p* = 0.045, after including age, sex, and BMI) predicted the proportion of changes in the HDL level, in patients after eradication treatment (Figure 2).

## Discussion

There are no previous studies that evaluate changes in microbiota related to lipid metabolism in otherwise healthy subjects pre-post-*H. pylori* eradication. Alterations in blood cholesterol levels (high LDL and low HDL) are major risk factors for metabolic syndrome and cardiovascular disease (17). Antibiotic treatment for *H. pylori* eradication increased the HDL, reaching values similar to controls, in agreement with previous studies (18), and both eradication and infection produced changes in the microbiota, data described in detail in our previous study (11). More interestingly, this is the first time that variations in blood lipid levels have been related to specific bacteria from the microbiome independent of age, sex, and BMI resulting *H. pylori* infection and eradication, suggesting that gut microbiota may affect specific aspects of lipid metabolism. Further, many of the identified taxa related to lipid metabolism are novel findings.

The use of broad spectrum antibiotics have been widely established to cause community-wide microbiota perturbations (19). In our study, *H. pylori* infection and its eradication with amoxicillin and clarithromycin for 10 days induced a dysbiosis affecting microbial diversity, something that has been associated to metabolic functions of intestinal bacteria. Low microbial functional richness has been associated to metabolic disease, including levels of fasting triglyceride, LDL and a raise in inflammation markers (20). In this sense, our study associated unfavorable lipid profiles (high LDL and low HDL level) with a low microbial richness in metabolically healthy subjects. In addition, this unfavorable lipid profile (lower HDL/LDL ratio) was associated with the phylum Bacteroidetes. As opposed, Firmicutes was associated with a favorable lipid profile (higher HDL/LDL ratio). The association between blood lipids and gut microbiome has been previously reported (2, 21). Firmicutes and Bacteroidetes are the two main bacteria phyla and they are implicated in the homeostasis of the host and fat accumulation. Interestingly, in our study, for the first time, Bacteroidetes and Firmicutes predicted HDL and LDL levels, regardless of age, sex, and BMI.

The raise in Bacteroidetes and the disminution in Firmicutes associated to the *H. pylori* infection and antibiotic-treatment could affect the production of key metabolites for the host, including SCFAs, bile acids, and lipopolysaccharide (LPS). In fact, acetate and propionate are produced mainly by members of Bacteroidetes while butyrate is typically produced by members of Firmicutes (22). Alterations in the microbiota as well as in their metabolites influence key processes as energy harvesting, the activation of the immune system, modulation of the chronic inflammation through the modification of the intestinal barrier permeability and perturbation of the reverse cholesterol transport, triggering in a higher susceptibility for some metabolic diseases (23).

The impact of the antibiotherapy, amoxicillin, and clarithromycin, on lipid metabolism as a result of the disturbance on the microbiota is an area of interest with limited knowledge at present. In this sense, we show a negative association between changes in the abundance of *P. stercorea* and HDL level and a positive association between changes in the abundance of *P. copri, Lachonobacterium, Delsufovibrio*, and HDL level at 2 months after completing antibiotic treatment.

*Lachnospiraceae* family has been specially associated with LDL (2) and development of metabolic disorders (24). Our study associated *Lachnospiraceae/Lachonobacterium* with HDL. *Lachnospiraceae* family is known by its capacity to metabolize complex polysaccharides to SCFAs, as butyrate, acetate, and propionate (25). Butyrate controls gene expression through epigenetic control. Buryrate is a key energy source for the colonic mucosa (26). Acetate is implicated in the *de novo* hepatic lipogenesis through acetyl-coA and fatty acid synthase (FAS), while propionate balances lipogenesis (27). Moreover, acetate is needed for the biosynthesis of cholesterol in liver. Thus, changes in the acetate/propionate ratio could play a crucial role in the regulation of lipid and cholesterol metabolism (28).

More interestingly is that alterations in *Delsufovibrio*, from the Proteobacteria phylum, and *Rikenellaceae*, from the Bacteroidetes phylum, predicted changes in HDL levels at 2 months after completing antibiotic treatment. To date, lower abundance of *Desulfovibrio* has been associated with obesity, blood pressure, insulin, and LDL (29). *Desulfovibrio* also has been inversely correlated to BMI (29). We showed, for the first time, that *Delsufovibrio* positively contributed to the HDL level independently of BMI, suggesting that this bacterium affects blood lipids partly. Bacteria such as *Delsufovibrio* and *P. copri* are producers of lipopolysaccharide (LPS). LPS is a ligand for toll-like receptor 4 (TLR4), which activates innate immune system, something that could trigger in a pro-inflammatory status (30). The inflammatory response to microbial stimulus may induce metabolic modifications. Low grade systemic inflammation associated with bacterial LPS-induced endotoxaemia as well as a gut permeability increase have been associated with elevated plasma HDL (31). This finding could be controversial, as obesity, metabolic syndrome and type-2 diabetes are characterized by and impaired barrier function and local systemic inflammation (32), and they are characterized by a low HDL. On the other hand, products secreted by Desulfovibrio can up-regulate CD36 expression (33). CD36 is a critical regulator of lipid absorption, which has been positively correlated with HDL (34). All of these works would be in accordance with our data, and could help explain the results obtained in this study. In addition, we have reported an inverse relationship between *Rikenellaceae*, glucose (AUC) (11) and HDL levels at 2 months antibiotic treatment. *Rikenellaceae* is able to use the environmental glucose for acetate production (35). Acetic acid stimulates “*de novo*” lipogenesis and cholesterogenesis in the liver (36). The increase in the production of SCFAs as well as the elimination of glucose from the environment by this bacterium could explain part of the observed results. In addition, tudies have been showed a possible role of *Clostridiales* (including *Lachonobacterium*) and *Bacteroidales* (including *Rikenellaceae, P. copri, and P. stercorea*) in the metabolism of lipids, through the metabolic pathway of bile acids (37). In fact, secondary bile acids from the bacteria metabolism are absorbed being able to systemically modulate lipid and glucose metabolisms through nuclear or G protein-coupled receptors (GPCRs), such as FXR or TGR5 (37).

The current study, although it has some strengths, there are also a few limitations to be considered. The targeted sequencing of the16S rRNA, although it permits know who is who, has the impediment to identify specific species and strains as well as gives little information on bacterial functions. Moreover, although previous sample size calculations were performed to ensure a real approach, sample size could be augmented. The lack of a group of subjects without an *H. pylori* infection exposed to the eradication treatment could be indicated as another limitation, although it was not performed because of ethical reasons. This group could have provided more detailed data on the role of antibiotic treatment. Lastly, the follow-up period was based on a visit at 2 months the administration of the antibiotic treatment. The introduction of new time-points after 2 months could permit us know more about the dynamics suffered after eradication treatment.

In summary, antibiotic eradication treatment for *H. pylori* could increase HDL levels and affected the gut microbiota composition. The results obtained indicate that intestinal microbiome, specifically bacteria such as *P. copri, Lachonobacterium* and *Delsufovibrio, Rikenellaceae* could plays a role in lipid metabolism.

## Data Availability Statement

The datasets generated for this study can be found in the SRA database public repository from NCBI within the BioProject accession number PRJNA517270.

## Ethics Statement

The studies involving human participants were reviewed and approved by Medical Ethics Committee at Virgen de la Victoria University Hospital and conducted in accordance with the Declaration of Helsinki. Written informed consent was provided by all participants, who also were verbally informed of the characteristics on the study. The patients/participants provided their written informed consent to participate in this study.

## Author Contributions

GM-N was person in charge of the metagenomic laboratory analysis, statistical analysis and interpretation of data, as well as the drafting, and reviewing of the manuscript. IC-P was person in charge of the recruitment of the patients and their follow-up and contributed to the design of the study and the revision of the manuscript. MR-R and JF-G also participated in the recruitment and follow-up of the patients, as well as reviewed the manuscript. MC-P performed laboratory analysis and participated in the revision of the manuscript. FC participated in the study design and reviewed the manuscript. IM-I assessed the bioinformatic analysis and critical revision of the manuscript. FT contributed to the study concept and design, interpretation of data, reviewed, and critically revised the article. All authors contributed to the article and approved the submitted version.

## Conflict of Interest

The authors declare that the research was conducted in the absence of any commercial or financial relationships that could be construed as a potential conflict of interest.

## Abbreviations

SCFAs: short-chain fatty acids
HDL: high density lipoproteins
LDL: low-density lipoproteins
OGTT: glucose tolerance test.
